# First-Year Training-Schools

**Published:** 1921-05-21

**Authors:** 


### THE MATRONS' AND SISTERS' DEPARTMENT.

### FIRST-YEAR TRAINING-SCHOOLS.

In devising plans for the co-ordination of all nurse-training establishments it has been usual to get over the difficulties connected with institutions not capable of giving full training by classing them as "preliminary schools." There is no doubt that many small institutions might be brought into line by this device, but it is plainly not suited to the fairly large number of training-schools which only just lack a sufficiency of beds, or some necessary class of patients, or other requisite to rank as full training-schools. Such are the Poor-Law infirmaries without a resident medical officer which at the present moment attempt to give a three-year certificate, can offer no prospect of "recognition" to their probationers, and are in consequence often in great straits for candidates.

In the admirable paper which Miss Seymour Yapp was unfortunately prevented from reading herself at the Nursing Conference she broached a plan which is worthy of trial in more than one direction. Her suggestion is that these imperfect or minor training-schools could be utilised for giving girls of nineteen or so two years' training in the first-year subjects recently put forth in the General Nursing Council Syllabus. She is confident, after wide experience of the resources of these infirmaries, that they possess facilities for giving this course, if spread over two years. The matrons are, for the most part, well-trained women, who take these posts as a step to promotion—and to make them a link in the training system of the country would, no doubt, add to the attractiveness of the work. The authorities, much harassed in advertising for candidates, would probably welcome any reasonable suggestion for adding to the teaching powers of the staff and raising the status of the probationers. Patients would benefit immeasurably from the increased interest aroused in their condition under the influence of a keen training-school, and the sole difficulty in the way of utilising this material for preparing candidates for registration lies in the problem of their future training.

If regarded solely as "Preliminary Schools," such institutions could offer their probationers nothing worth having for their two years' service. But if they ranked as "First-Year Schools," and if these first two years, spent in going through a well-planned course, availed, after examination, to pass the probationer into a larger infirmary as a second-year nurse for two more years, a very strong inducement would be offered to young women to enter the ranks of trained nurses by this portal. Miss Yapp has had personal experience in taking probationers with two years' training from small schools. Having passed the first-year nurses' examination, they took their place as second-year nurses, attended the senior lectures and demonstrations, and passed the final examination taken by an independent examiner. It was a great success. The nurses trained in the minor schools were swift to adapt themselves to the wider and different discipline necessary in the bigger establishment, and showed no inferiority. Once regulate the curriculum of the minor training-schools, and provide for adequate instruction from matron or sister-tutor (who need not in small schools devote her whole time to teaching), and the essentials of a good first-year's course can be guaranteed, even in the smaller institutions which have no resident medical officer.

The position of first-year probationers when they move on to the larger schools needs raising. Of course, in many respects they must at first rank as juniors and expect to do a good deal of routine work. But they need not be trampled on. Their status as second-year nurses should be secured, and the old exclusive attitude towards those not of the same brand must disappear. Miss Yapp's original and well-thought-out contribution towards the promotion of unity has the special value which comes from successful experimentation.

